# The Interface Between Reading and Handwriting

**DOI:** 10.3389/fpsyg.2022.892913

**Published:** 2022-07-06

**Authors:** Meredith Saletta Fitzgibbons

**Affiliations:** Speech-Language Pathology Program, Midwestern University, Downers Grove, IL, United States

**Keywords:** reading, handwriting, visual word form area, typical development, dyslexia, developmental coordination disorder

## Introduction

Zelaznik and Goffman (2010) state: “Language production, whether spoken, signed, or written, is a motor activity” (p. 383). There are specific connections between the linguistic process of reading and the motoric process of handwriting, including how they develop, where and how they are instantiated within the brain, and what can occur when their underlying neurological processes are compromised.

This connection is reflected in even the earliest research regarding disorders of reading and writing. Broadbent (1872), Berlin (1887), and Kussmaul (1887) first reported that acquired brain damage could cause patients to lose their speech, reading, and writing skills even when their non-verbal cognition was preserved. English physician Morgan (1896) and Scottish ophthalmologist Hinshelwood (1900) believed that medical problems caused difficulties in reading and writing. Researchers Myklebust and Johnson (1962) described symptoms which they observed in many of 200 children with dyslexia, including subclinical disturbances in motor coordination.

## The Development of Handwriting

Children learn to recognize letters before becoming able to write them (Reutzel et al., 2019). A child first begins to scribble with no discernible pattern (Coates and Coates, 2016). Between the ages of 2 and 3 years, he/she learns to imitate shapes (vertical strokes, horizontal strokes, and circles). The child may be ready to begin writing when he/she is able to cross the body's midline to imitate an oblique cross (Feder and Majnemer, 2007). Even at this young age, children use different arm and hand movement patterns when drawing as opposed to writing. McCutchen (2006) concludes that, to a certain extent, even these young children can distinguish between drawing and writing. By age four, children write marks arranged in a line with regular spacing (Scott, 2012). Researchers have learned about children's handwriting development from measures as diverse as examining their visual-motor integration abilities, kinematics of their pen movements, and eye movements as they write (Fears and Lockman, 2020). Researchers have also explored dynamic measures including the velocity, acceleration, pressure, and tilt of writers' pens (Gargot et al., 2020) and their pause behaviors (Sumner et al., 2013; Alamargot et al., 2020).

Throughout the early school years, learning handwriting contributes to the child's overall writing development (Graham et al., 2008). Learning stroke sequencing helps young people to memorize letter shapes (Longcamp et al., 2005; Mangen and Velay, 2010; Stevenson and Just, 2014)—a skill that keyboarding alone does not necessarily accomplish. Children also recognize letters more efficiently when writing than when typing them (Longcamp et al., 2005). A student's handwriting legibility can influence whether he/she qualifies for special education services (Graham et al., 2008; Cahill, 2009), ultimately impacting the child's motivation and self-esteem (Gargot et al., 2020).

## Reading and Handwriting in the Brain

Accounts of the ways in which both reading and handwriting are instantiated in the brain are remarkably similar. Both involve hierarchical sequences of processing. In their *Local Combination Detector Model*, Dehaene et al. (2005) describe how the brain processes increasingly complex combinations of visual features *en route* to reading. Kemmerer (2015) summarizes seven consecutive stages of processing in this “assembly line.” Processing begins when the retina of the eye perceives the visual stimulus and transmits information to the lateral geniculate nucleus of the thalamus. Here, cells process simple stimuli such as series of dots. Following this step, the brain's primary visual center in the occipital lobe is engaged. The occipital lobe integrates the representations of the thalamic cells to capture bars or lines of a variety of orientations; captures sections of contours or fragments of letters; recognizes entire letter shapes; supports invariant processing while ignoring superficial differences in font and case; and finally, processes bigrams (sequences of two letters) and quadrigrams (sequences of four letters).

Writing to dictation also follows a hierarchical sequence, in this case with five steps. First, when the ear hears the word, the brain recognizes the sound structure of the word. Next, the brain accesses the spelling of that word within the orthographic lexicon. Following that step, the *graphemic buffer*—a working memory system which temporarily holds the identities of the graphemes while the word is being written—is activated. Next, *allographic conversion* occurs, and the graphemes are translated from their abstract identities into concrete forms, depending upon whether the writer wishes to use print, cursive, uppercase, or lowercase letters. Subsequently, *graphomotor planning* occurs, and the brain instructs the motor system as to the size, direction, and sequence of movements to be executed. Finally, the motor act of handwriting occurs (Kemmerer, 2015).

The interface between reading and writing has its source in the brain. Reading and writing share some, but not all, neural substrates (Hillis, 2001; Tainturier and Rapp, 2001; Hillis and Rapp, 2004; Purcell et al., 2011). Within the cortex, the area most finely tuned for reading is the *visual word form area* (VWFA). This area is located within the lateral occipitotemporal sulcus and is lateralized to the left hemisphere. The VWFA may be activated by both reading and writing (especially for words with atypical spellings); however, the evidence from studies of brain injuries is equivocal on this point (Kemmerer, 2015). The dorsal premotor region facilitates both recognizing and writing letters (Velay and Longcamp, 2013). Exner's area is one of the main writing centers of the brain, and it is also activated during reading (Pattamadilok et al., 2016). The handwriting network described by Planton et al. (2013), including the supplementary motor area (SMA), pre-SMA, and putamen, is also activated during reading (Gosse et al., 2022). These findings indicate that writing actively facilitates fluent reading (Pattamadilok et al., 2016). As Kemmerer (2015) states, “when we see letters, our brains automatically recall the motor memories of how we manipulate a pen or pencil to produce them” (p. 219).

## What Clinicians Need to Know About the Interface

Individuals with reading differences/disorders including developmental dyslexia often experience persistent difficulties with handwriting (Sumner et al., 2016). Writing comprises the *central components* of language and orthography and the *motor/peripheral components* of execution (Planton et al., 2017). Gosse et al. (2022) used functional magnetic resonance imaging (fMRI) to examine the writing network in children with dyslexia. They discovered that these children experienced differences in cerebellar activation in both central and peripheral components. Thus, writing is not just a motor skill, as individuals with dyslexia may have difficulties in writing which are not directly caused by difficulties in graphomotor planning. Difficulties in handwriting may be related to difficulties in spelling, as students who struggle with spelling also tend to write more slowly and less fluently (Berninger et al., 2008; Hebert et al., 2018).

Dyslexia often does not occur in isolation but is comorbid with other neurodevelopmental differences such as developmental coordination disorder (DCD; Hill, 2001; Visser, 2003). Authors of the Diagnostic and Statistical Manual of Mental Disorders (DSM-5; American Psychiatric Association, 2013) state that DCD has four diagnostic criteria: clumsiness, slowness, or inaccuracy in performing motor skills; difficulties that interfere with activities of daily living; early onset of symptoms; and symptoms that are not explained by intellectual disability, vision problems, or another underlying neurological condition. Children with DCD may experience difficulties dressing themselves (e.g., buttoning shirts or tying shoelaces), eating with utensils and without mess, playing physical games, using tools such as rulers and scissors in school, and—most relevant to the topic at hand—producing legible handwriting (American Psychiatric Association, 2013). Motor difficulties in DCD may occur in either gross- or fine-motor skills (Harris et al., 2015).

There is a significant overlap between the experiences of individuals with language learning differences and individuals with DCD. Both may demonstrate weak hand lateralization and may be inclined to reach across their body's midline with their non-preferred hand (Hill et al., 1998). According to Hodgson and Hudson (2017), this is because unlike most adults with neurotypical development, individuals with neurodiversity may demonstrate altered hemispheric mapping and reduced left-hemisphere activation during speech production (Whitehouse and Bishop, 2008; Illingworth and Bishop, 2009). There are differences between these profiles as well. Berninger and Amtmann (2003) and Hebert et al. (2018) suggest that handwriting and spelling engage separate processes. Handwriting engages orthographic coding, whereas spelling engages both orthographic and phonological coding.

Professionals—including speech-language pathologists, school psychologists, and general and special education teachers—who work with individuals experiencing difficulties in reading, motor performance, or both, must be aware of several concepts. First, the task of writing is comparable to that of a juggler keeping many balls in the air simultaneously. These “balls” include planning one's thoughts, spelling, executing legible handwriting (McCutchen, 2006), and writing accurately. Often, the flight of one ball is sacrificed to keep the others aloft. Gombert (1992) describes this as a state of *cognitive overload*. Helping children to write more efficiently and automatically can allow them to direct their cognitive resources toward planning and generating ideas rather than dwelling on accurate spelling and handwriting (Wanzek et al., 2017). In clinical parlance, it is often beneficial to implement *benign neglect*. Rather than criticizing every aspect of the child's message, the clinician should focus on one aspect (e.g., grammar, spelling, handwriting, punctuation…) during a given lesson and neglect all other aspects to decrease the child's frustration. Concentrating on letter-writing fluency (LWF) as an instructional goal may be beneficial, as Reutzel et al. (2019) mention that LWF predicts academic success even in college students.

Clinicians may execute a range of strategies to utilize this interface to improve the learning outcomes of their clients. Specifically, multimodal practice involving the physical formation of letters can enhance reading outcomes. One treatment for reading employing multiple modalities and enjoying support in the literature is the Orton-Gillingham approach. In this approach, clinicians emphasize multisensory input, encouraging children to engage their visual, auditory, and kinesthetic/tactile learning pathways while breaking down reading into its individual components (Sayeski et al., 2019). The Montessori method, originally pioneered by Italian psychologist Maria Montessori, involves activities such as tracing letters cut from sandpaper, drawing letters in shaving cream, and conducting activities which combine vision and a sense of space (Dehaene, 2009). The Lindamood Phoneme Sequencing Program (Lindamood and Lindamood, 1998) also incorporates oral-motor, visual, and auditory feedback during teaching. The Wilson Reading System is another multisensory approach which includes instruction in phonemic awareness, phonics, fluency, vocabulary, and comprehension (Duff et al., 2015). Teachers using Elkonin boxes instruct children in phoneme segmentation and blending through the manipulation of tokens (Ross and Joseph, 2019). These are just some examples of therapeutic techniques that can be used to direct the client's attention to both reading and handwriting skills through the use of multiple modalities. Improvements in spelling can lead to improvements in handwriting and vice versa (Hebert et al., 2018).

The use of technology (including iPad applications) can improve handwriting legibility and speed (John and Renumol, 2018), as well as other visuomotor skills, in even very young children (Dessoye et al., 2017; Axford et al., 2018; Butler et al., 2019). Technology may be beneficial beyond traditional intervention approaches within occupational therapy, such as an emphasis on repetitive or multimodal practice (Zachry et al., 2020). Using technology can be particularly valuable because it provides convenience to teachers and motivation to students (Aronin and Floyd, 2013; Campigotto et al., 2013; Kaur, 2017; Zachry et al., 2020). Practice with technology can also improve other fine-motor outcomes including manual dexterity (Butler et al., 2019).

## Discussion

Reading and handwriting share similarities at many levels. Their developmental progression and their instantiation within the brain substantially overlap. Researchers and clinicians should be aware of symptoms that occur when underlying neurological processes are compromised as well as therapeutic techniques that can support the development of these two skills. The multifaceted interactions between these two modalities of language deserve further attention within the literature, especially concerning their practical implications in clinical settings.

## Author Contributions

The author confirms being the sole contributor of this work and has approved it for publication.

## Funding

This research was supported by the College of Health Sciences of Midwestern University, Downers Grove, IL, United States.

## Conflict of Interest

The author declares that the research was conducted in the absence of any commercial or financial relationships that could be construed as a potential conflict of interest.

## Publisher's Note

All claims expressed in this article are solely those of the authors and do not necessarily represent those of their affiliated organizations, or those of the publisher, the editors and the reviewers. Any product that may be evaluated in this article, or claim that may be made by its manufacturer, is not guaranteed or endorsed by the publisher.
